# The employment of art therapy to develop empathy and foster wellbeing for junior doctors in a palliative medicine rotation - a qualitative exploratory study on acceptability

**DOI:** 10.1186/s12904-024-01414-6

**Published:** 2024-04-01

**Authors:** Eng-Koon Ong, U-Tong Emily Tan, Min Chiam, Wen Shan Sim

**Affiliations:** 1 Division of Supportive and Palliative Care, National Cancer Centre Singapore, 30 Hospital Blvd, Singapore, 168583 Singapore; 2 Assisi Hospice, 832 Thomson Rd, Singapore, 574627 Singapore; 3 Duke-NUS Graduate Medical School, 8 College Rd, Singapore, 169857 Singapore; 4 Office of Medical Humanities, SingHealth Medicine Academic Clinical Programme, 31 Third Hospital Ave, Singapore, 168753 Singapore; 5 Division of Psycho-oncology, National Cancer Centre Singapore, 30 Hospital Blvd, Singapore, 168583 Singapore; 6 Division of Cancer Education, National Cancer Centre Singapore, 30 Hospital Blvd, Singapore, 168583 Singapore; 7 KK Women’s and Children’s Hospital, Singapore, 100 Bukit Timah Road, Singapore, 229899 Singapore

**Keywords:** Art therapy, Medical humanities, Medical education, Acceptability

## Abstract

**Background:**

The interdisciplinary realm of medical humanities explores narratives and experiences that can enhance medical education for physicians through perspective-taking and reflective practice. However, there is a gap in comprehension regarding its appropriateness at the postgraduate level, especially when utilising art therapists as faculty. This study aims to assess the acceptability of an innovative art therapy-focused educational initiative among junior doctors during a palliative care rotation, with the goal of cultivating empathy and promoting well-being.

**Methods:**

A qualitative research project was conducted at the Division of Supportive and Palliative Care (DSPC) in the National Cancer Centre Singapore (NCCS). The study involved the recruitment of junior doctors who had successfully completed a three-month palliative care rotation program, spanning from January 2020 to April 2021. In a single small-group session lasting 1.5 h, with 3 to 4 participants each time, the individuals participated in activities such as collage making, group reflection, and sharing of artistic creations. These sessions were facilitated by an accredited art therapist and a clinical psychologist, focusing on themes related to empathy and wellbeing. To assess the acceptability of the program, two individual interviews were conducted three months apart with each participant. An independent research assistant utilised a semi-structured question guide that considered affective attitude, burden, perceived effectiveness, coherence, and self-efficacy. Thematic analysis of the transcribed data was then employed to scrutinise the participants’ experiences.

**Results:**

A total of 20 individual interviews were completed with 11 participants. The three themes identified were lack of pre-existing knowledge of the humanities, promotors, and barriers to program acceptability.

**Conclusions:**

The participants have mixed perceptions of the program’s acceptability. While all completed the program in its entirety, the acceptability of the program is impeded by wider systemic factors such as service and manpower needs. It is vital to address these structural limitations as failing to do so risks skewing current ambivalence towards outright rejection of future endeavours to integrate humanities programs into medical education.

**Supplementary Information:**

The online version contains supplementary material available at 10.1186/s12904-024-01414-6.

## Introduction

The medical humanities (MH) may be defined as a field that uses the humanities’ intricate and sympathetic knowledge about the human condition and its ability to examine particularistic and experiential knowledge to help ensure a morally sensitive, narratively sound, and deeply professional clinical practice [1, 2]. In medical education, content and pedagogy from the arts and humanities are integrated into the teaching and learning of medical students, trainees, and practicing physicians. These elements support the mastery of skills, reflective practice, and transformative growth [2–11]. Since 2018, we have initiated pilot studies aimed at integrating content and methods from the visual arts, literature, and theatre to cultivate self-awareness and empathy among junior doctors specializing in palliative care (PC) [2, 5–6]. These initiatives have undergone refinement based on frameworks proposed by review papers, advocating for increased collaboration among various stakeholders in the arts and humanities [3, 12]. 

## Current gaps

Despite indications of acceptability and effectiveness, the presence of mixed feedback, low participation rates, difficulties in program evaluation, and a dearth of qualitative data from research on the arts and humanities in medical education have highlighted the need for a more in-depth exploration of the integration of medical humanities (MH) in medical education [1, 5, 6, 12]. The considerations involved in the designing and implementation of a medical humanities program into the curriculum deserve greater attention as they have direct bearings on the selection of faculty for such programs [13–16]. For instance, our clinician-educators (CEs) could employ medical education methodologies in conjunction with biomedical and psychotherapeutic concepts, ensuring relevance during these programs [14]. However, CEs frequently express their lack of training in the analytical methods of the MH as a barrier towards employing more in-depth methods such as close reading, narrative inquiry, and conversational analysis [17–19]. Conversely, humanities scholars and artists may face scepticism from learners within medical education programs due to their perceived lack of patient contact [1]. In the light of these challenges, and after contemplating a broader range of faculty options, we identified art therapy as a feasible avenue in our humanities program [20–24]. Art therapists (ATs), equipped with training in both the arts and clinical practice, play a crucial role in bridging potential gaps in ideologies, curricular implementation, and research between medical education and the humanities [29–30].

Art therapy can be defined as a form of therapy that effectively addresses individual and relational treatment goals, as well as community-related concerns. It is utilised to enhance cognitive and sensorimotor functions, nurture self-esteem and self-awareness, build emotional resilience, foster insight, improve social skills, alleviate conflicts and distress, and contribute to societal and ecological change. This versatility allows for its application in both clinical practice and the promotion of staff wellbeing [25–30]. With stressors associated with the pandemic coming to a fore, there was a heightened urgency to explore the incorporation of art therapy in supporting the well-being of our junior doctors.

## Context

This study was undertaken at the Division of Supportive and Palliative Care (DSPC) within the National Cancer Centre Singapore (NCCS). The DSPC functions as a specialised palliative care (PC) service offering consultation for patients with life-limiting illnesses. These patients are either admitted to the affiliated Singapore General Hospital (SGH) or seen in outpatient specialist clinics. Clinical teams, comprising social workers and art therapists (ATs), provide PC through various means such as ward rounds, interdisciplinary case discussions, and informal consultations [31]. 

Within three PC teams, each consisting of a specialist consultant, a senior resident or resident physician, and a PC nurse, junior doctors in their postgraduate years two to five, with varying experience in PC, perform comprehensive PC assessments. They are involved in symptom management, facilitating complex serious illness conversations, and offering psychosocial and grief support to patients and their families. However, due to the inherent clinical complexities associated with PC, further exacerbated by pandemic-related segregation measures and limited manpower, junior doctors have faced challenges in managing their daily clinical responsibilities [23, 32–39]. Recognizing the potential of art therapy to address the urgent need for support, a novel art therapy-focused program has been integrated into the existing orientation curriculum for all junior doctors since 2020. This program is implemented over the first month of their rotation, emphasizing empathy development and fostering well-being. The aim of this study is to evaluate the acceptability of the program among junior doctors.

## Research questions

In view of the nature of the program as a pilot educational intervention, we wanted to first assess its acceptability. The following research questions were formulated for this study: “What were the junior doctors’ experiences when participating in this novel art therapy-based program?”, “What impact did the program have on empathy and wellbeing?” and, specifically, “How acceptable was the program as perceived by the participants?”

## Program format and materials

The study team comprised of two physicians (OEK, SWS), an accredited art therapist (ET), and a humanities scholar (CM). ET played a crucial role in designing the program and facilitating the sessions. The program was conducted quarterly, featuring a single session lasting 1.5 h co-facilitated by ET and a clinical psychologist. Materials, including magazines, paper boxes, scissors, glue, and colouring and writing stationary were provided. During the session, participants were prompted to reflect on a clinical encounter that had left an impact on them as they browsed through images and words in the provided magazines. With the assistance of guiding questions, participants selected images that resonated with them and used them to create collages, both within and on the outside of the paper boxes provided. Collages within the box were meant to reflect the possible emotions of various stakeholders involved in the clinical encounters, while those on the outside illustrated the behaviors of different parties. Subsequently, participants shared their thoughts behind their creations with the rest of the group, with each group consisting of four to five participants.

The structure of the session drew inspiration from the principles of art therapy, particularly the use of collages and boxes. These artistic mediums were chosen intentionally to facilitate the expression and exploration of personal experiences, emotions, and perspectives among the participants. The incorporation of collages and boxes allowed for a creative and symbolic representation of the clinical encounters, enriching the participants’ engagement and expression within the therapeutic process [35–44]. 

The utilisation of images from magazines played a pivotal role in guiding participants to reflect on their experiences, while simultaneously easing the anxiety and pressure associated with creating art from scratch. These images served as powerful prompts, allowing for multiple layers of symbolic and metaphorical representation and providing avenues for the narration of clinical encounters. The process of selecting and collaging these images may also have had a cathartic effect for some participants. The incorporation of the box as a physical representation of space served to safeguard the expression of complex and diverse emotions. This stood in contrast to the collages, which illustrated the behaviours of patients and caregivers on the external surface of the box. Together, these elements served as a reminder to participants that there is often a deeper layer of context and emotions underpinning certain behaviours. This facilitated the process of meaning-making and enhanced self-awareness among the participants.

Our study also draws upon Schon’s work on reflective practice [45]. The education framework outlined here emphasises that decision-making is shaped not only by previous curriculum and learned experiences but also often occurs “on-the-go,” where quick decisions are crucial to prevent both morbidity and mortality. Our program facilitates reflection “on-action” as participants recall clinical encounters that left a lasting impression during their clinical practice. The intention is that when confronted with similar situations and challenges in the future, participants will engage in reflection “in-action.” This involves internalizing the insights gained during the session and making informed judgments based on the lessons learned.

The interviews were conducted at two distinct timepoints to coincide with participants transitioning to other clinical postings, along with the use of ongoing elicited interim research texts, to enable both interviewers and participants to continually construct narratives based on their lived experiences within the temporal and spatial contexts. This approach aligns with the theory of reflective practice, emphasizing that past experiences within the program continue to prompt profound reflections even after participants have concluded their palliative medicine posting.

The program was also crafted based on several key principles. Firstly, it was designed to welcome junior doctors with no prior experience in art-making, ensuring that any lack of artistic background did not deter participation. Secondly, the chosen instructions and methods were structured to create a safe and reassuring environment, allowing participants to share their thoughts at their own pace and comfort levels. To enhance psychological safety, ground rules highlighting the significance of mutual respect, confidentiality, and the option to leave the session if needed were communicated before the start of each session. The lesson plan for our program is outlined in Appendix I.

## Methods

### Participant recruitment

All forty-two junior doctors, comprising both medical officers and residents, who had completed the art therapy-based program between January 2020 and April 2021 during their rotations, were invited to participate in this study without regard to their age, gender, or years of training. Doctors on elective rotations, those of the grade resident physician or senior resident and above, as well as other team members such as nurses, social workers, and medical students, were excluded from the study.

Invitations were initially sent via email by the principal investigator, along with attachments of the participant information sheet (PIS) and consent form. The PIS outlined the study’s purpose, recruitment inclusion criteria, the process involving two individual interviews conducted by an independent research assistant (RA) lasting no more than an hour each, the potential risks and benefits of participation, and the provision of a ten-dollar retail voucher for each completed interview. The significance of the study and the assurance of participant anonymity were emphasised. Interested individuals would respond to the email and arrange to meet the RA for the consent-taking process. A reminder email was sent two weeks later, and for non-responses, no further contact was made. After obtaining consent, the RA provided details about the individual interviews.

### Data collection

The first interview took place within two months of the participants completing the program, while the second interview occurred within three months after the conclusion of their palliative care (PC) rotation. A questionnaire, comprising both prompting and probing questions, was developed. This questionnaire was designed in accordance with Sekhon et al’s acceptability framework, which centers on affective attitude, burden, perceived effectiveness, coherence, and self-efficacy (see Appendix II) [46]. Given that the concept of medical humanities might not be familiar to the participants, we offered the definition provided by Shapiro et al., as stated in the introduction above.

Due to safety restrictions imposed by the pandemic, all interviews were conducted electronically and audio recorded. The recorded data was later transcribed by the RA. The interviews had no set time limit and concluded when there was no further input from the participants. Each participant was assigned a number (e.g., P1, P2) by the RA to anonymise the data for subsequent analysis by the study team. Recruitment of participants ceased when the co-authors (OEK, CM, SWS) determined that data saturation had been reached, and no new themes were emerging.

### Data analysis

The data was analysed using thematic analysis, a qualitative research method focused on identifying, analyzing, and reporting patterns within the data [47]. This method not only organises and describes the data in rich detail but also interprets various aspects of the research topics. Guided by the principles of reflexive thematic analysis, the analysis was carried out collaboratively by the three co-authors (OEK, CM, SWS) and encompassed the following six phases: familiarisation of the data, initial code generation, prototype themes construction, potential themes review, themes definition, and reporting of themes.

The data analysis commenced with each co-author independently creating familiarisation notes. Analysing transcripts with an open mind, points of interest were noted, and initial codes were generated, guided by the research question [48]. This process involved collating points in the familiarization notes to reflect and interpret participants’ shared experiences. The list of codes was reviewed during the creation of a code book, and initial themes were developed to consolidate common understanding and patterns within the codes, anchored by main concepts. Through an iterative process involving repeated discussions between the co-authors, an early thematic map and subsequently a finalised thematic map were created, using descriptive and concise names. Additionally, incomplete data from prematurely terminated interviews were also analysed.

### Rigour of data collection and analysis

Individual interviews were chosen over focused group discussions due to the personal nature of the artwork created during the sessions. This decision is aimed at promoting a safe and secure environment for participants to share their experiences and allowed for a more in-depth exploration of the rich and personal aspects of their engagement in the program [49]. 

The RA took steps to minimise concerns over faculty biases, peer judgement, and potential impact on their appraisals and reputation and was trained by the study team members to highlight these considerations. Participants were reassured that they could halt the interviews at any point if they felt uncomfortable without the need for detailed explanations. The study team also provided the RA with contextual information about the local healthcare and training systems, ethical considerations, and reinforced desirable facilitation techniques such as open-ended questioning and maintaining a non-judgmental stance.

To refine the interview questions, the questionnaire was piloted with two junior doctors, identifying challenges and guiding improvements [50]. All data were returned to the participants to ensure the accuracy of transcription. The co-authors EK and CM possessed postgraduate Masters’ training in medical education and the medical humanities. Consensus was reached before concluding recruitment, driven by data saturation. Themes and subthemes were derived through repeated cycles of iterative discussions.

## Results

A total of eleven participants were recruited, and 20 interviews were conducted, with each session lasting an average of 45 min. Eleven interviews were conducted at timepoint 1, and nine at timepoint 2. The analysis revealed three main themes: lack of pre-existing knowledge of the humanities, promotors, and barriers to program acceptability.

### Lack of pre-existing knowledge of the humanities

Most participants had limited knowledge about the humanities with little prior experience. The humanities were conflated with activities like *“art and craft”* (P11), *“distraction”* (P1), ethics (P6), religion (P4, 6), and *“subjects studied in secondary schools like geography”* (P9). The responses suggest an association of the humanities with recreation, fun, and activities outside the realm of traditional medical education. Adding to these uncertainties, participants perceived existing humanities events, such as concerts and newsletters, as *“not particularly… obviously related to our practice of medicine”* (P9, P11). This lack of pre-existing knowledge of the humanities led to questions about “*what to do, what are you doing, what are some of the expectations, what is the kind of agenda…”* (P4) at the start of the novel art therapy-based program. However, all participants completed the program and they expressed in the interviews that the art-making and small-group sharing sessions were safe spaces that provided peer support, and participants found them to be generally pleasant experiences (P1,2).

### Promotors of program acceptability

The participants recognised the impact of the art making on reflective practice and promoting self-awareness. The participants suggest that the positive impact led to greater empathy and ability to connect with patients and peers, resulting in improved satisfaction with care.

#### Promoting self-reflection

The program is intentionally designed to provide adequate time and space for reflection. The opportunities to reflect on challenging clinical encounters, process emotions, and develop self-awareness were described as rare and treasured by several participants. The dedicated time for reflection allowed them to *“capture emotion or a feeling that you may not be able to express verbally… made us pause to think…”* (P8), *“consolidate my experiences…”* (P1), and *“gain inspiration”* (P7), and was deemed *“cathartic”* (P11). Some participants appreciated a deeper meaning in their work, particularly its humanistic aspect, acknowledging that it *“isn’t just about the numbers…”* (P9). Participants also recognised the value of understanding how their peers were coping which created a sense of shared emotions and mutual support: *“It was useful to know how my peers are doing and that many of what we feel when we see our patients are shared emotions… my fellow colleagues were also experiencing the same thing…”* (P2).

The nature of art-making was particularly highlighted as helpful in breaking down barriers, with one participant noting that *“it bridges that kind of barrier because if you… ask people to sit around and talk about their experience, who will really open up?”* (P3). Through the arts-based program, participants also developed empathy for both oneself and others and learned to cope better with work stressors as expressed by one participant: *“think about what else I can do… removes part of the stress…”* (P6). Additionally, participants appreciated hearing different perspectives and strategies, with one participant noting, *“it’s good to hear different perspectives and what they did in that situation which may help you… facing something similar… to try what the people have already done”* (P9).

#### Professional development

Through the art-making experience, participants recognized the importance of taking an empathetic approach towards patient care. More significantly, participants were able to identify the ways in which empathetic care is achieved as a result of the activities. Participants felt that the program allowed them to become “*more well-rounded and holistic… and meet them(patients) on (a) more common ground”* (P8). The capacity to empathise was seen as crucial, as *“when doctors explore a patient’s needs”* (P3), *“relate [to them]… [possess] the wisdom [as to] how to talk to them…”* (P4) and understand *“why people have certain diseases and why certain health behaviours happen”* (P11), the likelihood of prescribing effective treatment increased.

Despite recognising the value of the program, many participants rejected the idea that assessments be embedded into the program (P2,6,7). They expressed concerns about safety and honest sharing which might even lead to a more stressful experience of the program.

### Barriers to program acceptability

While the participants observed positive role-modelling where empathetic patients care *“from the top down”* was practiced *(P1, 7–10), o*ur analysis reveals that the acceptability of the program is challenged by personal and systemic factors. At the individual level, participants were concerned that *“some may be more linguistically inclined…or relate better to music” (P1)* instead of art and thus would be *“a bit stressed” (P1) and* become *“too task-oriented” (P7)*. Participants also highlighted the lack of time and competing service needs as being particularly inhibiting. For instance, P4 commented that it was *“pressurising… at the back of my mind I still have all these things I have to do by the end of the day”*. These barriers led to doubts about the effectiveness of the program (P3, P5).

## Discussion

The data analysis has revealed mixed responses to the study’s research questions on the experience of the participants and the acceptability of the program. While the lack of awareness of the humanities did not hinder completion of the program since its relevance gradually became apparent to the participants, significant barriers exist. Specifically, ***individual*** and ***systemic*** factors warrant further exploration.

Individually, the lack of previous exposure and understanding of the humanities generated uncertainty regarding the expectations, value, and relevance of the program, leading to a shared sense of doubt and anxiety amongst the participants even though the junior doctors approached the program with openness [1, 51, 52]. The diversity of individual preferences creates an additional layer of challenge as their acceptability of the program was contingent upon compatibility between the artmaking and their spiritual and religious beliefs [53], learning styles [54], preferences for activities of leisure, and coping styles [55, 56]. 

Many participants also expressed reluctance towards the inclusion of formal assessments in the arts therapy-based program. The participants raised concerns that formal evaluations would impede honest and safe sharing for fear of “failing” the subject [57]. Yet, medical education places great emphasis on measuring quantifiable outcomes and formal assessments to “drive learning” [58–61]. Educational programs without formal evaluations in medical education are immediately perceived as informal, ad hoc, and therefore, have no significant impact on training, appraisal, and indeed personal wellbeing [62]. Therein lies a common struggle many educators face in their endeavours to integrate the humanities in medical education where they needed to illustrate the legitimacy of programs with embedded assessments without compromising the learners’ acceptability of the program [63, 64]. 

Systemic factors appear to further skew our participants from ambivalence towards outright rejection of the program. Firstly, the humanities program had to be designed and standardised such that it is capable of training multiple batches of junior doctors in quick succession due to the frequent and short rotations of junior doctors into the speciality. Due to the prioritization of mass applicability of the program, the diverse beliefs and values of different participants may not have been adequately addressed in a single session [21, 65–67]. Secondly, an expected mandatory attendance despite overwhelming service needs led to significant distress as doctors struggled with anxiety and guilt about neglecting their patients [68, 69]. Finally, without the time and opportunity to build trust and relationships amongst peers and seniors due to the brevity of clinical rotations, the experiences of the program could not be reinforced or reflected upon beyond the program itself. This could have reduced the willingness of the junior doctors to invest in time and effort on the program and by extension, the program acceptability.

### Limitations

The single site of recruitment may limit the generalisability of results across institutions. Due to the low enrolment rates, we could not identify other factors that may be associated with program acceptability such as prior undergraduate education in the humanities, differing perceptions according to seniority and training experience, impact of previous and current levels of wellbeing [70]. However, it is also possible that the data itself may explain the poor recruitment rates due to systemic pressures to prioritise service over participation in research [71]. Finally, this study pivots on the retrospective views of participants on the program which may have been coloured by recall bias.

## Conclusion

Existing research studies at the intersection of medical humanities and medical education have largely focused on elucidating the epistemological functions and assessing learning outcomes of the integration of the humanities into medical education curricula [72–81]. While numerous articles have put forth suggestions to support the integration of the arts and humanities in medical education [82], there is a gap in the scholarship on the success rates of programs looking to ***operationalise*** such an endeavour. Our data highlights the need to consider both individual learners’ preferences and wider systemic factors.

### Electronic supplementary material

Below is the link to the electronic supplementary material.

Supplementary Material 1

Supplementary Material 2

## Data Availability

The datasets used and/or analysed during the current study are available from the corresponding author on reasonable request.
