# Supplementary material for: The employment of art therapy to develop empathy and foster wellbeing for junior doctors in a palliative medicine rotation - a qualitative exploratory study on acceptability
Source: BMC Palliat Care. 2024 Apr 1;23:84. doi: 10.1186/s12904-024-01414-6 (PMC10983679; doi:10.1186/s12904-024-01414-6)
Supplement: Supplementary file 1 — Supplementary Material 1 [file 12904_2024_1414_MOESM1_ESM.docx]

Appendix I

Lesson plan of educational program

| **Duration** | **Contents** |
| --- | --- |
| 10mins | Introduction to the session and the space  Sharing ground rules |
| 20mins | Art Directive I *(working with the box base)*  1. Think of a case experience that has impacted you.   - Select 3-5 images (from the pile of magazine images provided) that best portray the interactions/ behavior/ characteristics of the patient and/or caregivers and your responses to the person(s).   *Prompters:*  *Select images that “jump” out to you from the pile.*  *Key characteristics can be:*  - *Profile of the patient, family, significant others*  - *Interactions e.g. patient vs family, patient/family vs healthcare team, patient/family vs you*  -  *What did you have to do?*  -  *What did you dread/ avoid doing?*  -  *What did you find yourself doing more?*   - Place the selected images on the outside of the box.     2. If you look into the box and the inside box is how the patient/caregiver and you feel inside, what would it look like?   - Select another 3-5 images that best depict how you think the patient/family member/ significant others and yourself might be feeling during those interactions and when they exhibited those behaviors. |
| 30mins | Sharing   - Take some time to look at your box and share with us the case experience you have selected.   *Prompters: - What makes you choose this particular case experience?*  *- How is it like for you to relook at this experience in this manner?* |
| 15mins | Art Directive II *(working with the box cover)*   - On the exterior of the box cover, select another 3-5 images that would depict how you might do/ respond differently should you have similar case.   *Prompters:*  *As we talk about your case experience, I wonder if there is anything you might do/response differently if you have a similar case again or for any other cases to come.* |
| 15mins | 1. Final sharing  *Prompters: -  How has this particular case experience influence your practice?*  *-  Does empathy mean no boundaries?*  *-  Now that you’re ending your posting, what does empathy look like to you?*  *- Can you bring empathy to your next posting?*    2. Closing *(the interior of the box cover)*   - You are encouraged to take out this box maybe six months or 1 year later and look at it again, especially the cover that reflects what you may do differently. - You can complete the interior of the box cover as you ask yourself, “have I done differently?” “how do I feel about how being able/ not able to do it differently now?” |
