# Supplementary material for: The employment of art therapy to develop empathy and foster wellbeing for junior doctors in a palliative medicine rotation - a qualitative exploratory study on acceptability
Source: BMC Palliat Care. 2024 Apr 1;23:84. doi: 10.1186/s12904-024-01414-6 (PMC10983679; doi:10.1186/s12904-024-01414-6)
Supplement: Supplementary file 2 — Supplementary Material 2 [file 12904_2024_1414_MOESM2_ESM.docx]

| **Prompt** | **Probe** |
| --- | --- |
| **Qn 1. Please share with me your experiences in attending the Medical Humanities sessions.** | |
| - What about the session left an impression on you? - What do you remember about attending the session? | - Why do you think this left an impression on you? - Was this a positive or negative experience? - How did you felt when you participated in the session? |
| **Qn 2. What comes to your mind when you hear “medical humanities”?**  **- How do you feel about the humanities being used as a teaching tool?** | |
| - Provide the definition of medical humanities: The medical humanities can be described as *the use of methods, concepts and content from disciplines like literature, art and poetry to investigate illness and inform healthcare professionals how to better understand their professional identity with the aim of increasing self-awareness and improving humanistic care.* - Is this the first time you have attended a medical humanities session? | - Why do you feel as such? - What do you think are the advantages of using the humanities? - What do you think are the disadvantages of using the humanities? |
| **Qn 3. How do you think humanities can be used in medical education?** | |
| - What do you think is the intention of using humanities programs (in education)? - Do you think such programs would develop your professional qualities as a doctor?   - Empathy, compassion, patient-centred care and communication with patients. | - What can healthcare professionals learn from humanities? - Why do you think it is important to develop such professional qualities as a doctor? |
| **Qn 4. How would such programs influence your personal life?** | |
| - Do you think the humanities are relevant to you outside of your professional work? - Do you think this program has helped you develop any personal qualities? | - Why do you think so? - Could you share some examples? |
| **Qn 5. What are your thoughts if participants who have attended the Medical Humanities session go through an assessment?** | |
| *Assessment of participants’ understanding and application of humanities.   - Do you think it is appropriate? - Do you think assessments are essential? - Do you think the program would impact all the participants equally? | - What would make a good or bad assessment tool? - How would the presence of assessment affect your participation in such programs? - What will influence the acceptability/participation in the program? |
| **Qn 6. Would you be interested in participating in more projects based on the humanities in the future? *(Closing)*** | |
| - What more would you hope to see in future medical humanities session? - Would you like to share any feedback with regards to the medical humanities programme? - Is there anything else you would like to share? | |

| **Prompt** | | **Probe** |
| --- | --- | --- |
| **Qn 1. Do you remember your experience in the HAPPE sessions?** | | |
| - Do you remember what you did during the HAPPE sessions? - Do you remember what you took away from the HAPPE session? | | - Have there been any changes to your understanding of “medical humanities”? - Do you think participation in the HAPPE sessions left an impression on you even after you left the posting? |
| **Qn 2. Has there been any changes in your clinical practice that is influenced by your participation in HAPPE?**  **- As a doctor, do you see any changes in how you perceive your clinical work** | | |
| - Did participation in the HAPPE sessions influence how you practise medicine eg communications, clinical reasoning, interprofessional collaboration and communications? | | - Do you think that you are reminded about empathy and patient-centred care when you think about HAPPE? - Does that improve your empathy levels in clinical care? |
| **Qn 3. How do you cope or manage stress nowadays?**  **- Have there been any stressful instance during your clinical work?** | | |
| - Did participation in the HAPPE sessions help you cope with stress better? - Do you think humanities program can be used to teach doctors how to cope with stress? | | - Do you use the humanities to cope with stress? Eg literature, art, music, etc. - If no/yes – why/how so? |
| **Qn 4. Do you think humanities can be used in medical education?** | | |
| - Having been through the HAPPE program and DSPC posting for more than 3 months now, what do you think about humanities being used as a teaching tool now? - Do you think such programs are essential in your medical education as a doctor?   - Empathy, compassion, patient-centred care and communication with patients. | | - Do you think the humanities are a good tool to use in medical education? |
| **Qn 5. In the future, how would you prefer your classes or programs based on the humanities to be structed?** | | |
| - What delivery methods would you prefer?   - class, discussion, reflective writing - What medium would you prefer?   - movies, songs, stories, poetry - What setting would you prefer?   - online, group, individual - What more would you hope to see in future medical humanities session? | - Why do you prefer such mode? - Why do you prefer such medium? - Why do you prefer this setting? - Why do you think that is lacking? / Why is that something that you hope to see? | |
| **Closing** | | |
| - Would you like to share any feedback with regards to the medical humanities or medical humanities programme? - Is there anything else you would like to share? | | |
